# [Corrigendum] Screening for implicated genes in colorectal cancer using whole-genome gene expression profiling

**DOI:** 10.3892/mmr.2025.13768

**Published:** 2025-12-04

**Authors:** Long-Ci Sun, Hai-Xin Qian

Mol Med Rep 17: 8260–8268, 2018; DOI: 10.3892/mmr.2018.8862

Subsequently to the publication of this paper, an interested reader drew to the authors’ attention that, in the “*Identification of hub genes for CRC*” subsection of the Results on p. 8263, the left-hand column, in the first sentence the reference to Matthew's correlation coefficient algorithm should perhaps have been written as the Maximal Clique Centrality algorithm. The authors have replied to confirm that, upon carefully reviewing the paper, the Maximal Clique Centrality algorithm from the CytoHubba plugin was indeed used to identify the top 20 hub genes, and that, during the manuscript preparation, the full name of “MCC” was incorrectly written as “Matthews correlation coefficient” due to an oversight on their part. Therefore, the first sentence in this subsection of the Results section should have read as follows: “To identify potential hub genes among the 306 genes previously identified, **the Maximal Clique Centrality (MCC) algorithm from the CytoHubba software plug-in was used.**”

The authors sincerely apologize for any confusion or misunderstanding this error may have caused for the readers, and are grateful to the Editor of *Molecular Medicine Reports* for granting them the opportunity to publish this corrigendum.

